# Involvement of Disperse Repetitive Sequences in Wheat/Rye Genome Adjustment

**DOI:** 10.3390/ijms13078549

**Published:** 2012-07-10

**Authors:** Diana Tomás, Miguel Bento, Wanda Viegas, Manuela Silva

**Affiliations:** Centro de Botânica Aplicada à Agricultura, Secção de Genética, Instituto Superior de Agronomia, Technical University of Lisbon, Tapada da Ajuda, Lisboa 1349-017, Portugal; E-Mails: dianarstomas@isa.utl.pt (D.T.); miguelmbento@isa.utl.pt (M.B.); wandaviegas@isa.utl.pt (W.V.)

**Keywords:** genome adjustment, triticale, wheat-rye addition lines, dispersed repetitive sequences, pSc20H

## Abstract

The union of different genomes in the same nucleus frequently results in hybrid genotypes with improved genome plasticity related to both genome remodeling events and changes in gene expression. Most modern cereal crops are polyploid species. Triticale, synthesized by the cross between wheat and rye, constitutes an excellent model to study polyploidization functional implications. We intend to attain a deeper knowledge of dispersed repetitive sequence involvement in parental genome reshuffle in triticale and in wheat-rye addition lines that have the entire wheat genome plus each rye chromosome pair. Through Random Amplified Polymorphic DNA (RAPD) analysis with OPH20 10-mer primer we unraveled clear alterations corresponding to the loss of specific bands from both parental genomes. Moreover, the sequential nature of those events was revealed by the increased absence of rye-origin bands in wheat-rye addition lines in comparison with triticale. Remodeled band sequencing revealed that both repetitive and coding genome domains are affected in wheat-rye hybrid genotypes. Additionally, the amplification and sequencing of pSc20H internal segments showed that the disappearance of parental bands may result from restricted sequence alterations and unraveled the involvement of wheat/rye related repetitive sequences in genome adjustment needed for hybrid plant stabilization.

## 1. Introduction

Repetitive DNA sequences have been extensively studied in large plant genomes, corresponding up to 83% and 92% of *Triticum aestivum* and *Secale cereale* genomes, respectively [1]. These interspersed repetitive sequences correspond mainly to transposable elements that are ubiquitous in all organisms and represent a considerable genome fraction, particularly in plants with large genomes [2]. Repetitive sequence fraction is strongly implicated in functional genome structure [3], and transposable elements have been successfully used as molecular tools to characterize complex plant genomes [4–7]. Furthermore, the elucidation of mechanisms underlying parental adjustment in hybrid genotypes has also revealed the role of transposable elements in genome merger induced restructuring [8], particularly in Triticeae hybrids and polyploids species, such as distinct synthetic wheats and triticales (reviewed in [9]).

In polyploids, where more than two basic sets of chromosomes share the same nucleus, an irreversible outbreak of paternal genome reorganization has been described in several plant systems [10]. Polyploidization is suggested to have occurred spontaneously in 30%–70% of plant species, an assessment that reaches almost 100% if paleopolyploids are considered [11,12]. Moreover, polyploidization constitutes the starting point to synthetic introgression of alien chromatin in plant crops like wheat. The allopolyploid triticale (×*Triticosecale*), synthesized through the hybridization of wheat (*Triticum* spp.) and rye (*S. cereale*), has been broadly used in the production of wheat-rye addition, substitution, and translocation lines by backcrossing to wheat [13]. The involvement of transposable-related sequences in polyploidization induced genome evolution in the wheat-rye system was evaluated through Inter Retrotransposons Amplified Polymorphism (IRAP) and Retrotransposons Microsatellite Amplified Polymorphism (REMAP) methodologies using primers designed for LTR regions of barley (*Hordeum vulgare* L.) [14,15]. IRAP and REMAP analysis was crucial to disclose genomic modifications accessed by alterations in banding profiles, corresponding mainly to rye parental genome band losses [9,15]. Moreover, it was suggested by Fluorescent *In Situ* Hybridization (FISH) experiments that those methodologies target dispersed sequences preferentially clustered in rye sub-telomeric chromosome domains [15].

The presence of rye chromatin in wheat background has been detected through RAPD analysis [6,16]. The screening of 413 random 10-mer primers performed by Francis *et al.* [16] identified a rye genome-specific marker, the sequence of which was afterwards published as pSc20H rye-genome cloned marker [6]. pSc20H is a 1494 bp sequence related to retrotransposons of several plant species like sorghum, rice, pineapple, and Arabidopsis, dispersed throughout rye chromosomes, except at telomeric and nucleolar organizing regions [6,16]. Several rye-specific repetitive sequences, including pSc20H, have recently been pointed out as excellent tools to assess evolutive patterns of rye species [17]. We have previously disclosed the strong involvement of retrotransposon and microsatellite flanking sequences mainly clustered in sub-telomeric domains in genome restructuring in the wheat-rye system. Now, we intend to assess the involvement of pSc20H-like dispersed sequences in genome rearrangements induced in triticale and wheat-rye addition lines.

## 2. Results

OPH20 RAPD marker and pSc20H sequence [6] were used to evaluate hybrid genome rearrangements and identify sequences involved in wheat-rye genotypes adjustment in octoploid triticale and in the seven wheat-rye addition lines. These wheat addition lines are composed of a hexaploid wheat genome plus a single pair of each rye homologous chromosomes. The high inbred nature of the same wheat, rye and triticale lines analyzed in this work has been extensively demonstrated previously using several molecular markers [14,15] as well as through chromosome constitution confirmation of all the wheat-rye addition lines used [14].

### 2.1. OPH20 10-mer Primer PCR Analysis

**Figure 1:**
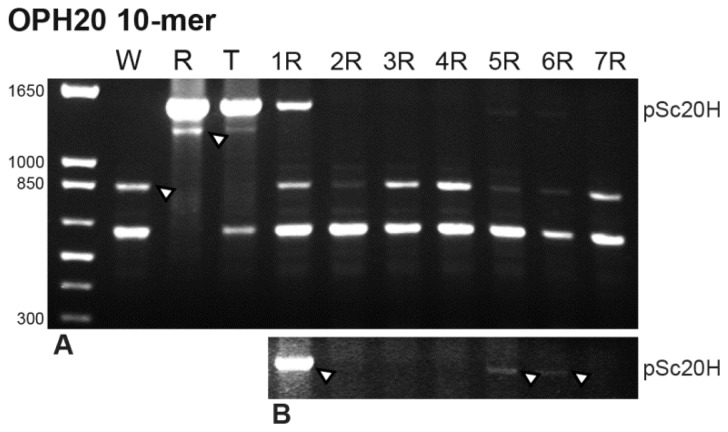
OPH20 10-mer primer PCR banding profiles of wheat (W), rye (R), triticale (T) and seven wheat-rye addition lines (numbers correspond to rye homologous chromosomes pair). (**A**) Arrows indicate a wheat-origin band absent in triticale but present in all wheat-rye addition lines and a rye-origin band present in triticale but absent in all addition lines. (**B**) An overexposed copy of **A**, where arrows indicate pSc20H band presence only in 1R, 5R and 6R wheat-rye addition lines. Molecular weight marker: 1 Kb Plus DNA Ladder.

RAPD marker obtained with the OPH20 10-mer primer [16] (Experimental Section) produced characteristic and reproducible banding profiles between 500 bp and 1650 bp in all species analyzed (Figure 1). Minor and non-reproducible bands were not considered in the banding profile analysis performed. The results obtained are summarized in Table 1. In the rye genome, two fragments were amplified, one with the expected ~1500 bp and another with 1300 bp. In wheat, the two rye-characteristic bands referred to previously were not present and two distinct bands, of ~650 bp and 850 bp, were observed.

The triticale banding profile was compared with wheat and rye parental lines and with wheat-rye addition lines produced by controlled backcrossing of octoploid triticale to hexaploid wheat followed by self-fertilization. The analysis of triticale banding profile showed two characteristic rye-origin bands and only the ~650 bp wheat-origin band. The wheat-origin ~850 bp band is consistently absent in the triticale banding profile (Figure 1). The analysis of wheat lines with the addition of each pair of rye chromosomes revealed the presence of the two wheat-origin bands in all lines analyzed. Concerning rye-origin bands, the ~1300 bp band is absent in all wheat addition lines and the ~1500 bp band (pSc20H) is only present in wheat addition lines comprehending 1R, 5R and 6R rye chromosomes (Figure 1B).

All bands observed in wheat and rye parental lines using OPH20, except the rye-origin ~1500 bp band previously sequenced in rye cv. Imperial (pSc20H, [6]), were gel-isolated, purified and cloned for sequence analysis. The sequences obtained were analyzed though NCBI nucleotide alignment.

Rye-origin ~1300 sequence (OPH20Rye1300, accession number JX120155) BLAST alignment revealed 87% homology with the following published sequences: *Aegilops tauschii* clone BAC RI43D6 hypothetical protein and plastid acetyl-CoA carboxylase (Acc-1) genes (EU660897.1, [18]); *A. tauschii* HMW-glutenin locus (AF497474.1, [19]) within a retrotransposon cluster region; and *T. aestivum* clone BAC 1067B03 (EU835982.1, [20]) in a *copia*-like retrotransposon sequence.

Wheat-characteristic larger sequence (OPH20Wheat850, accession number JX120156) NCBI nucleotide alignment revealed 86% sequence similarity with only one published sequence, a *T. aestivum* chromosome 3B-specific BAC library contig ctg0464b (FN564430.1, [21]), covering a 173 bp segment of the LTR of *gypsy*-type retrotransposon RLG_Romani. The other wheat-origin sequence of ~650 bp (OPH20Wheat650, accession number JX120157) revealed 97% sequence similarity with *T. aestivum* cv. Chinese Spring clone BAC 400N24 containing a *TaCKX2.3* cytokinin oxidase/dehydrogenase gene that was mapped to chromosome 3D (JF292901.1, [22]). OPH20Wheat650 sequence also shares 90% homology with *T. aestivum* chromosome 3B-specific BAC library contig ctg0464b (FN564430.1, [21]) within Erika *gypsy*-type retrotransposon.

Moreover, a pSc20H sequence (accession number AF305943) BLAST revealed a sequence similarity between 83% and 97% with several retrotransposon *gypsy*-like partial sequences isolated from distinct *Secale* species (*S. cereale*, *S. vavilovii*, *S. strictum*, *S. sylvestre*) recently published [17]. Rye origin 1494 bp sequence also revealed between 81% and 79% sequence similarity with several *Triticum* sp. loci: 81% with *T. aestivum* chromosome 3B-specific BAC library, contig ctg0091b (FN564428.1, [21]); 80% with *T. turgidum* A genome HMW glutenin A gene locus (AY494981.1, [23]); 80% with *T. aestivum* BAC clones (DQ537335.1, [23]); and 79% with *T. aestivum* clone BAC D5 (EF426565.1, [24]).

### 2.2. pSc20H Sequence Analysis

**Figure 2:**
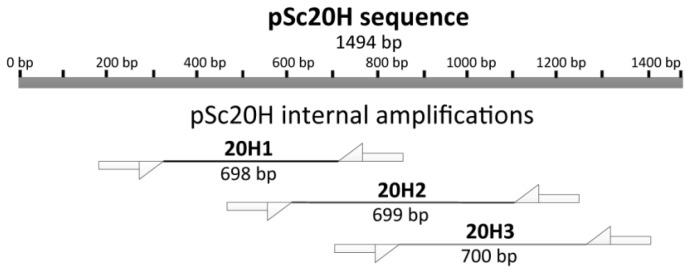
Dimensions (bp) of fragments expected from the amplification of three internal segments of pSc20H (accession number AF305943): 20H1, 20H2, and 20H3.

Differences between wheat-rye addition lines for amplification products using OPH20 10-mer primer were detected based on the presence or absence of pSc20H band. Thus, we further delineated a deeper analysis of such sequences using three pairs of primers designed to amplify pSc20H internal segments, namely 20H1 (698 bp); 20H2 (699 bp); and 20H3 (700 bp) (Figure 2).

**Figure 3:**
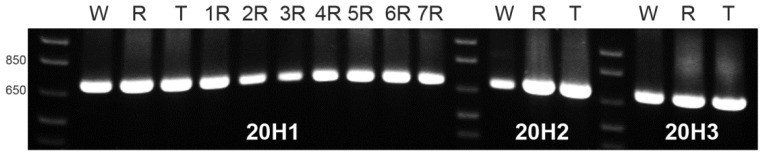
PCR banding profiles of wheat (W), rye (R), triticale (T) and seven wheat-rye addition lines (numbers correspond to rye homologous chromosomes pair) obtained with primers designed to amplify the three internal segments of pSc20H: 20H1, 20H2 and 20H3. All the pSc20H internal primers amplified a band with the expected size and identical in wheat, rye and triticale. Segment 20H1 is also amplified in all wheat-rye addition lines. Molecular weight marker: 1 Kb Plus DNA Ladder.

Identical bands with the expected size for the three referred pSc20H internal segments were amplified from wheat, rye and triticale. Moreover, using DNA from all wheat-rye addition lines as template, similar bands were obtained for the 20H1 segment (Figure 3) as well as for 20H2 and 20H3 segments (results not showed). Thus, although the rye-origin ~1500 bp (pSc20H) band is not present in wheat and wheat lines with the addition of rye chromosomes 2R, 3R, 4R, and 7R, amplified sequences with the expected size were obtained for the three internal fragments for all genotypes.

DNA dilutions used as templates in the initial OPH20 10-mer PCR experiment and in the PCR reactions using primers to the pSc20H internal segments were virtually the same. Therefore, our results clearly show that although pSc20H rye-origin ~1500 band is not amplified in wheat and in wheat lines with the addition of 2R, 3R, 4R and 7R rye chromosomes, all internal fragments of pSc20H, covering a total extension of 1226 bp are present both in wheat and in the whole set of wheat-rye addition lines.

Bands amplified from wheat, rye and 1R, 3R, 4R 5R, 6R, and 7R addition lines using primers for 20H1 internal segment of pSc20H sequence were gel-isolated, purified and cloned for sequence analysis. Sequences amplified from different clones obtained from the same band revealed 100% homology with each other.

The 20H1 sequence amplified from rye (20H1Rye, accession number JX120158) shares 94% sequence similarity with the expected segment of pSc20H (pSc20H_20H1) and 20H1 sequences amplified from wheat addition lines of 1R, 5R and 6R rye chromosomes share 97% (20H1 1R, accession number JX120160), 95% (20H1 5R, accession number JX120163) and 96% 20H1 6R, accession number JX120164) homology with rye 20H1 sequence, respectively. 20H1 sequences amplified from the three wheat-rye addition lines referred to above share a level of sequence similarity between 96% and 98% with each other.

Wheat 20H1 sequence (20H1Wheat, accession number JX120159) shares only 81% sequence similarity with the expected pSc20H segment (pSc20H_20H1) and 83% with 20H1 sequence amplified from rye. BLAST nucleotide alignment of the wheat 20H1 sequence revealed 95% homology with *T. aestivum* cv. Chinese Spring clone BAC 400N24 containing *TaCKX2.3* cytokinin oxidase/dehydrogenase gene (JF292901.1, [22]) in a region adjacent to the one that shares homology with OPH20Wheat650. Moreover, Wheat 20H1 shares between 96% and 100% sequence similarity with the equivalent sequences amplified from 3R, 4R, and 7R wheat addition lines (accession numbers JX120161, JX120162 and JX120165, respectively).

**Figure 4:**
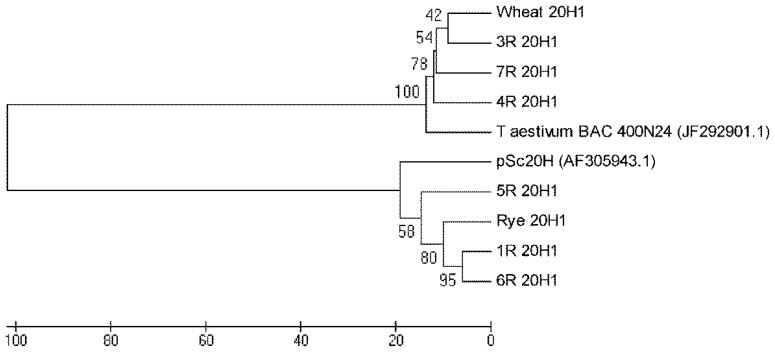
Neighbor-joining tree constructed utilizing the method [25] based on nucleotide alignment of 20H1 internal segments amplified from wheat, rye and wheat-rye addition lines. The numbers on the branches represent bootstrap support for 1000 replicates. The scale indicates the percentage of divergence. Sequences were aligned using Mega software version 5.05 [26] Accession numbers: pSc20H sequence (AF305943) and *T. aestivum* BAC 400N24 (JF292901.1).

The alignment of 20H1 sequences is presented in Supplementary Material Figure S1 for rye and wheat addition lines 1R, 5R and 6R and in Figure S2 for wheat and wheat addition lines 3R, 4R and 7R. The phylogenetic tree constructed for 20H1 sequences presented in Figure 4 clearly shows their separation into two major clusters. One cluster comprehends 20H1 from wheat and wheat addition lines 3R, 4R and 7R as well as *T. aestivum* BAC 400N24. The other one encompasses 20H1 from rye and wheat addition lines 1R, 5R and 6R as well as pSc20H.

## 3. Discussion

Although it is presently widely established that polyploidization leads to the appearance of new phenotypes and eventually to the creation of new species with higher plasticity [27], a deeper knowledge concerning parental genome adjustment in hybrid genotypes is still imperative. Wheat-rye synthetic genotypes with a distinct genome constitution and the respective parental lines constitute exceptional models to understand parental genome remodeling in hybrid genotypes. Previous REMAP and IRAP analysis of those genotypes, targeting mainly dispersed sequences preferentially clustered in sub-telomeric domains, proved the occurrence of a high level of genome rearrangements involving predominantly rye parental genome [14,15]. In the present work we intend to evaluate the involvement of rye-characteristic sequences dispersed throughout all rye chromosomes with the exception of sub-telomeric regions [6,16] in genome restructuring events induced by polyploidization in the wheat-rye system. We used OPH20 10-mer primer selected by [16] to analyze genotypes with wheat genome plus distinct rye genome compositions, namely triticale and wheat-rye addition lines, in comparison with their wheat and rye parental lines. The results obtained show that OPH10 primer yield distinct banding profiles in wheat and rye species. Distinct profiles of parental lines allow an accurate assessment of parental genome remodeling in hybrid genotypes involving 75% of the total number of bands produced.

Regarding wheat-origin bands, in triticale only OPH20Wheat650 is present, with the OPH20Wheat850 wheat-specific band being absent. However both bands are present in all wheat-rye addition lines, which is thought to result from a backcross of triticale to wheat involved in the production of those lines. On the contrary, the two bands amplified in rye are present in triticale, being however polymorphic in wheat-rye addition lines: OPH20Rye1300 sequence is absent in all the wheat-rye addition lines and pSc20H-like 1500 bp sequences are amplified in three of the seven lines. Although not referred to by the authors, in [6] a OPH20Rye1300 similar band is evident in several rye and triticale lines analyzed that also seems to be absent in wheat-rye addition lines analyzed by those authors [6]. Concerning the 1500 bp band, even though Francis *et al.* [16] and Ko *et al.* [6] suggested pSc20H sequence as a rye-specific marker, Botez *et al.* [28] did not obtain amplification of pSc20H-like sequences in all wheat lines with rye introgression.

The results that we attained confirm parental genome restructuring shown mainly in the disappearance of bands observed in progenitor profiles as previously described through Amplified Fragment Length Polymorphism (AFLP) analysis [29,30] as well as through IRAP, REMAP and Inter Simple Sequence Repeat (ISSR) [14,15]. Moreover, our results reinforce the dynamic nature of sequence restructuring induced by genome engineering in the wheat-rye system. In fact, in triticale a wheat-characteristic sequence is lost as a result of wheat-rye genome merger and subsequently rye-origin sequences are affected in wheat-rye addition lines. Likewise, a similar phenomenon was previously described concerning retrotransposon/microsatellite flanking sequences since wheat bands absent in triticale are present in wheat-rye addition lines [14]. Conversely, RAPD analysis revealed that rye-origin bands are increasingly affected by the backcross of triticale with wheat and further auto-fertilization of their progeny, as previously suggested [14].

The molecular tools used not only constitute an original approach to detect sequence rearrangements induced by polyploidization, but yield also novel outcomes concerning the analysis of specific sequences affected by genome merger. In fact, sequence analysis of bands amplified from wheat with OPH20 primer revealed sequence similarity with both repetitive and coding loci. The wheat restructured sequence absent in triticale (OPH20Wheat850) revealed sequence similarities with a *gypsy*-type retrotransposon LTR sequence [21]. A rye-specific sequence (OPH20Rye1300), absent in all wheat-rye addition lines, shares homology with distinct *Aegilops* loci containing d-genome HMW-glutenin [19] and acetyl-CoA carboxylase (Acc-1) genes [18] as well as with *copia*-like retrotransposon sequences of a *T. aestivum* [20]. The assessed genome rearrangement events are not restricted to non-coding regions of plant genomes, supporting the suggestion of Vitte and Panaud [31], stating that large plant genomes include extensive heterochromatin blocks mainly composed of retrotransposons interspersed with gene-rich regions. Moreover, the remodeling of both genome fractions has been suggested to be involved in genome redundancy which needs to be overcome for different genomes to adjust to a hybrid nucleus [32].

To further examine the extension of parental genome remodeling affecting pSc20H, three pairs of primers designed to amplify distinct internal overlapped segments (20H1, 20H2 and 20H3) were used. Unexpectedly, all internal sequences were amplified on all genotypes analyzed including the ones where pSc20H-like sequences were not amplified (wheat and 2R, 3R, 4R and 7R wheat-rye addition lines). Such results demonstrate that differences concerning pSc20H amplification observed in the present study as well as in previous ones [6,16] may result from restricted differences involving OPH20 10-mer primer annealing sites. This proposal is corroborated by several similar results formerly reported. Sequence-specific amplified polymorphism (SSAP) analysis with distinct anchored primers to pSc20H sequence revealed that not all primer combinations used resulted in the rye-specific bands [33]. Agreeably, in a previous work only one from several different combinations of primers designed to pSc20H sequence yielded a rye-specific band, the others combinations amplified sequences from both wheat-rye genotypes as well as from wheat [34]. Additionally, 20H1 sequencing analysis disclosed distinct sequences amplified from rye and wheat genomes. Rye-origin 20H1 sequence is highly homologous to the target pSc20H_20H1. Contrastingly, wheat-origin 20H1 sequence revealed high sequence similarity with *T. aestivum* a 3D-specific BAC containing a TaCKX2.3 cytokinin oxidase/dehydrogenase gene (OsCKX2). This gene was reported to affect yield in rice and was recently characterized in wheat [22]. Thus, wheat 20H1 restructuring suggests functional implications of distinct genome union in hybrid genotypes. Those inferences must be considered in plant breeding programs aiming to develop cereal crops with increased productivity traits.

Additionally, the 20H1 sequences phylogenetic tree presented in Figure 4 shows a clear separation into two major clusters. One cluster comprehends sequences amplified from rye and wheat addition lines presenting pSc20H sequences (1R, 5R and 6R) and shares high homology levels with pSc20H. The other cluster encompasses 20H1 sequences amplified from wheat and wheat addition lines where pSc20H is absent (3R, 4R and 7R) and shares a high sequence similarity with *T. aestivum* BAC containing the OsCKX2 gene locus [22]. Thus, the analysis of 20H1 internal sequences clearly shows that the markers used in this study target distinct but related sequence families present in retrotransposon-rich genome regions. The sequence similarity levels of 80% observed between sequences from the two major clusters moreover confirm the phylogenetic proximity of wheat and rye species.

## 4. Experimental Section

### 4.1. Plant Material and DNA Isolation

The following plant materials were used: hexaploid wheat *T. aestivum* L. “Chinese Spring” (2*n* = 6*x* = 42, AABBDD), diploid rye *S. cereale* L. “Imperial” (2*n* = 2*x* = 14, RR), the correspondent synthetic octoploid triticale (*T. aestivum* “Chinese Spring” × *S. cereale* “Imperial”; 2*n* = 8*x* = 56, AABBDDRR), and a set of seven wheat-rye addition lines, each composed of the entire hexaploid wheat genome plus a single pair of rye homologous chromosomes. Wheat-rye addition lines were produced through controlled backcrossing of the octoploid triticale to the wheat parent, followed by successive generations of self-fertilization and subsequent rye chromosome selection. The rye cultivar “Imperial” used is highly inbred and the octoploid triticale and their corresponding wheat-rye addition lines are at least 35 generations old [29]. All seeds used in the present study were from the original E.R. Sears seed stocks and were obtained from the USDA-Sears collection, Columbia, MO, USA.

Seeds from all genotypes were germinated and grown in controlled conditions at a 16 h light (20 °C)/8 h dark (20 °C) cycle. Genomic DNA was isolated from fresh young leaves of 8-week-old plants using modified cetyltrimethylammonium-bromide (CTAB) method [35]. The presence of a pair of rye chromosomes in each wheat-rye addition line was confirmed using GISH with rye total genomic DNA as well as through PCR amplification of pSc200, a rye-origin sub-telomeric tandem repeat (accession number Z50039) [14].

### 4.2. PCR Amplification, Electrophoresis and Data Analysis

PCR analysis was performed using OPH20 10-mer as primer (Table 2), a RAPD marker firstly described by [16]. Twenty-five microliter PCR reactions were performed with 1× PCR buffer, 2.5 mM MgCl_2_, 0.8 mM dNTP’s, 1.6 μM primer, 1.5 U Taq polymerase, 20 μg BSA, 10 ng DNA template and the following program: 5 min at 94 °C; 45 cycles of 1 min at 94 °C, 1 min at 36 °C, and 2 min at 72 °C; and a final 5-min extension at 72 °C.

To further characterize the pSc20H specific sequence, it was amplified using the referred OPH20 10-mer primer (accession number AF305943, [6]), three pairs of primers were designed to amplify three internal segments of that sequence (Table 2, Figure 2). Twenty microliter PCR reactions were performed with 1× PCR buffer, 1.5 mM MgCl_2_, 0.25 mM dNTP’s, 1 mM each primer, 0.5 U Taq polymerase, 50 ng DNA template and the following program: 5 min 94 °C, 30 cycles of 45 s 94 °C, 45 s 60 °C and 1 min of 72 °C, and a final extension of 5 min at 72 °C.

PCR products were run on 1.7% agarose gels using as molecular weight marker 1 Kb Plus DNA Ladder (Invitrogene), detected by ethidium bromide and photographed using a BIO-Rad GEL DOC 2000. OPH20 10-mer PCR and pSc20H internal segments amplification banding profiles presented (Figures 1,3) were consistently obtained in three technical replicates for each PCR experiment being therefore considered reproducible patterns for each specific reaction mixture. Selected bands were gel isolated, purified using High Pure PCR Product Purification Kit (Roche), cloned using TOPO^®^ TA Cloning^®^ Kit in pCR2.1 vector and sequenced. The sequences obtained were analyzed through BLAST nucleotide alignment on NCBI and sequence similarity between them was verified using BioEdit version 7.1.3.0 sequence alignment editor (Ibis Biosciences, California). Phylogenetic and molecular evolutionary analyses were conducted using MEGA version 5 [26].

## 5. Conclusions

RAPD methodology using OPH20 10-mer marker initially developed by Freancis *et al*. [16] to achieve a rye-specific molecular marker, revealed an additionally promising approach to understand parental genome modulation in hybrid genotypes. Both parental-origin sequences were affected by hybridization. However, rye-origin sequences are preferentially remodeled in wheat-rye addition lines. Thus, genotypes where rye chromatin is present in lesser amounts show more remodeling, as previously proposed [14]. The absence of the rye-origin sequence in wheat-rye addition lines seems to result essentially from the restructuring of restricted DNA sequences encompassing primer annealing sites. Furthermore, the analysis of pSc20H internal sequences shows that the markers used in this study target distinct wheat and rye-origin sequences. However, those sequences belong to related families present in retrotransposon-rich genome regions, confirming the phylogenetic proximity between wheat and rye. On the other hand, both dispersed repetitive sequences preferentially clustered in rye subtelomeric regions [15] and rye sequences uniformly distributed in other chromosome domains [6,16] seem to be involved in parental genome adjustment. The results presented will certainly contribute to a solid comprehension of plant hybrid genome plasticity highlighting the role of genome repetitive fractions. Moreover, this work supports the involvement of genome regions encompassing retrotransposons interspersed with coding sequences in the parental adjustment needed to stabilize polyploid species.

## Supplementary Information



## Figures and Tables

**Table 1:** Characterization of PCR banding profiles obtained with OPH20 10-mer primer in wheat, rye, triticale and wheat lines with the addition of rye chromosomes.

Number of bands in distinct genotypes profiles					

Parental lines		Hybrid lines			
	
		Triticale		Addition lines	
			
Wheat	Rye	Expected	Observed	Wheat + 1R, 5R, 6R	Wheat + 2R, 3R, 4R, 7R
2	2	4	3	3	2
OPH20Wheat850	pSc20H		pSc20H	pSc20H	OPH20Wheat85
OPH20Wheat650	OPH20Rye1300		OPH20Rye1300	OPH20Wheat850	OPH20Wheat650
			OPH20Wheat650	OPH20Wheat650	

**Parental origin**					
Wheat		2	1	2	2
Rye		2	2	1	0

Note: In triticale restructuring events are revealed by the difference between the number of observed bands and the number of expected ones (corresponding to the sum of the number of bands detected in parental lines). In wheat-rye addition lines, two groups presenting distinct banding profiles are characterized.

**Table 2:** Primers used for PCR analysis.

Primer	Sequence
OPH20 10-mer	5′-GGGAGACATC-3′
20H1	
For	5′-TGTCAAAAGCCAAATCACGA-3′
Rev	5′-CGTTGATGTTCTGCTTTCCA-3′
20H2	
For	5′-ATTTCATGCCGAAGGAGATG-3′
Rev	5′-GCAATCCCATGTTTCACCTT-3′
20H3	
For	5′-TGGGAAGAAAGTGAGCGACT-3′
Rev	5′-CATCTCGACCCAACGAGTCT-3′
