# Supplementary material for: Involvement of Disperse Repetitive Sequences in Wheat/Rye Genome Adjustment
Source: Int J Mol Sci. 2012 Jul 10;13(7):8549–61. doi: 10.3390/ijms13078549 (PMC3430250; doi:10.3390/ijms13078549)
Supplement: Supplementary file 1 [file ijms-13-08549-s001.pdf]

# Involvement of Disperse Repetitive Sequences in Wheat/Rye Genome Adjustment

## Supplementary Information

**Figure S1.** 20H1 Alignment. Full multiple alignments obtained with BioEdit of the expected segment of pSc20H (pSc20H_20H1) and 20H1 internal sequence amplified from rye and wheat-rye addition lines 1R, 5R and 6R.

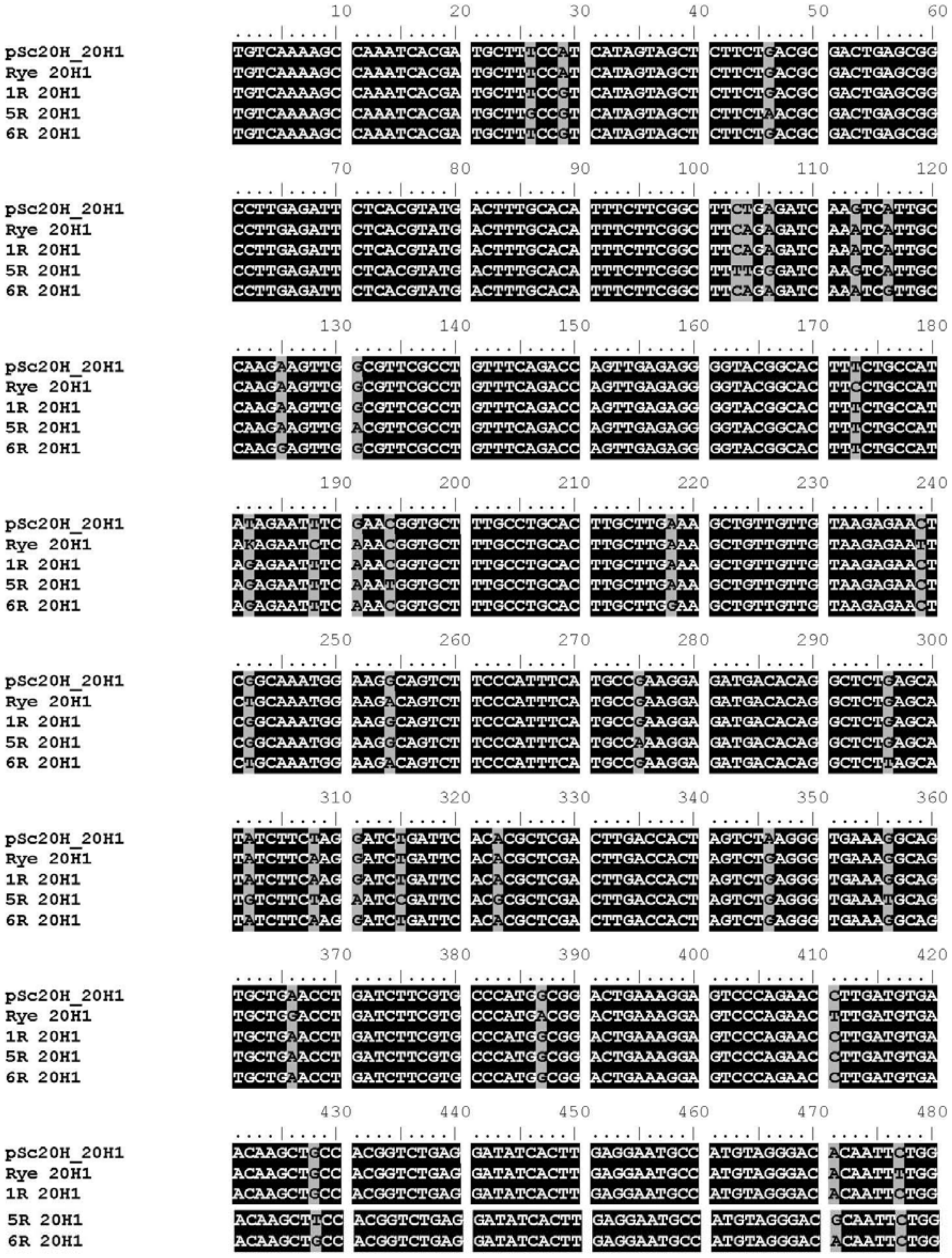

**Figure S1.** *Cont.*

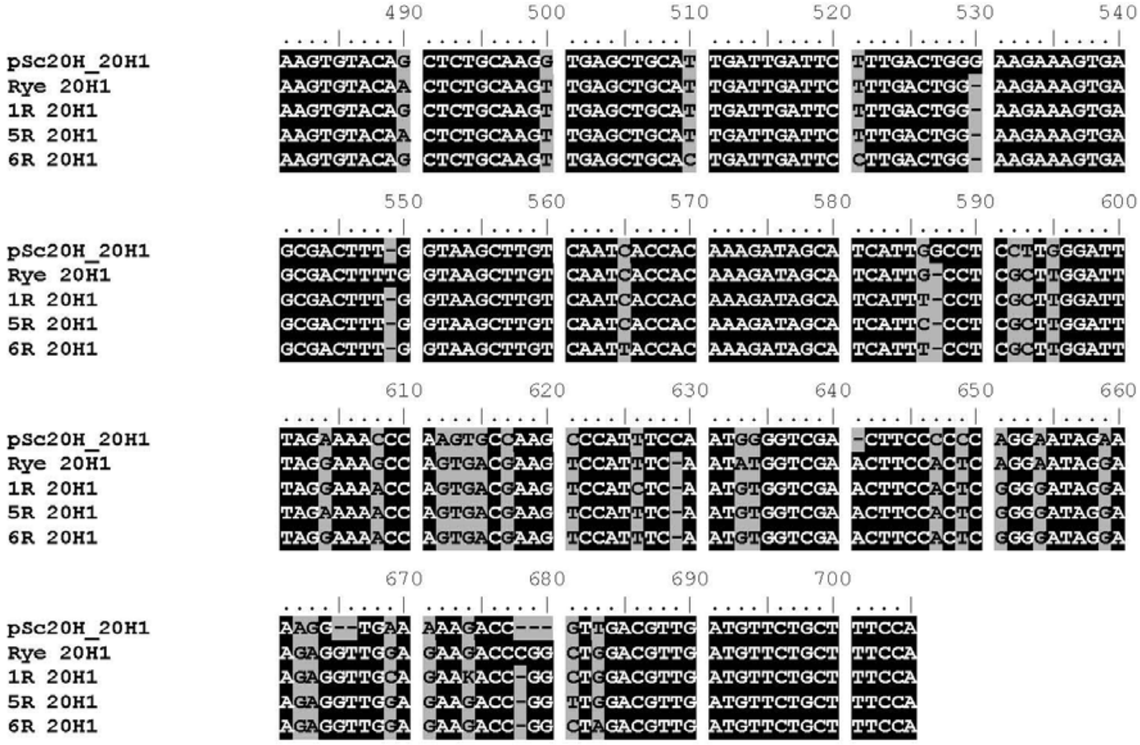


**Figure S2.** 20H1 Alignment. Full multiple alignments obtained with BioEdit of 20H1 internal sequence amplified from wheat and wheat-rye addition lines 3R, 4R and 7R and *T.aestivum* BAC400N24 (JF292901.1).

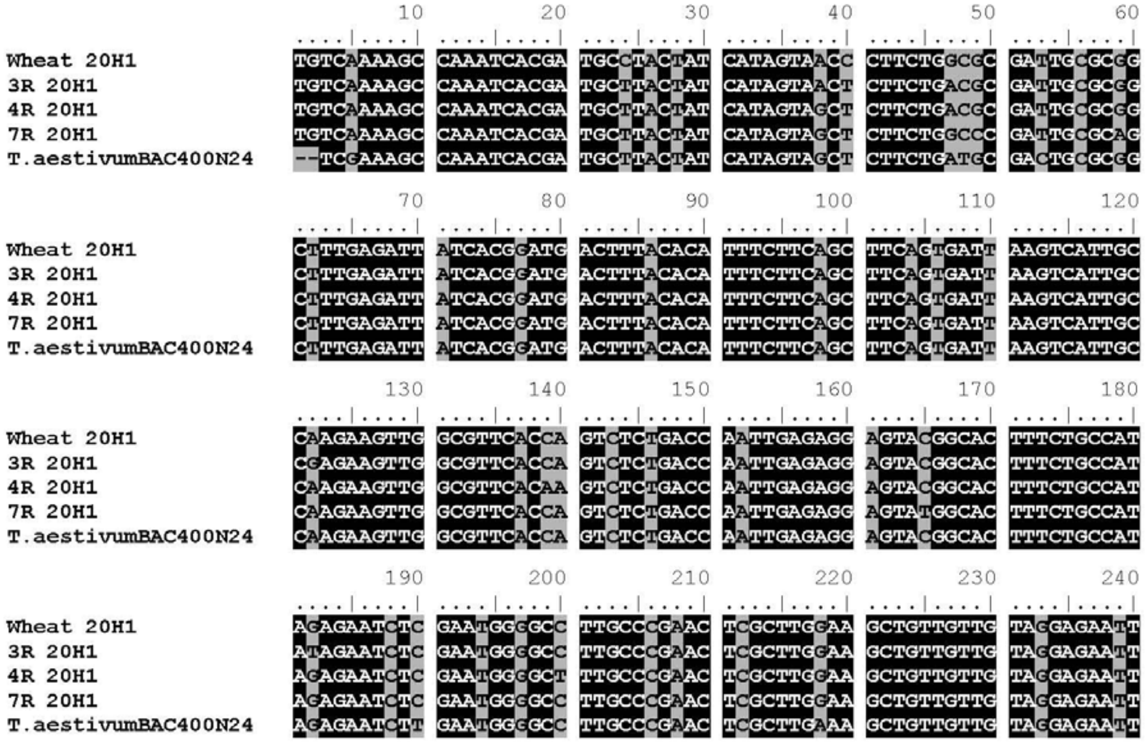

**Figure S2.** *Cont.*

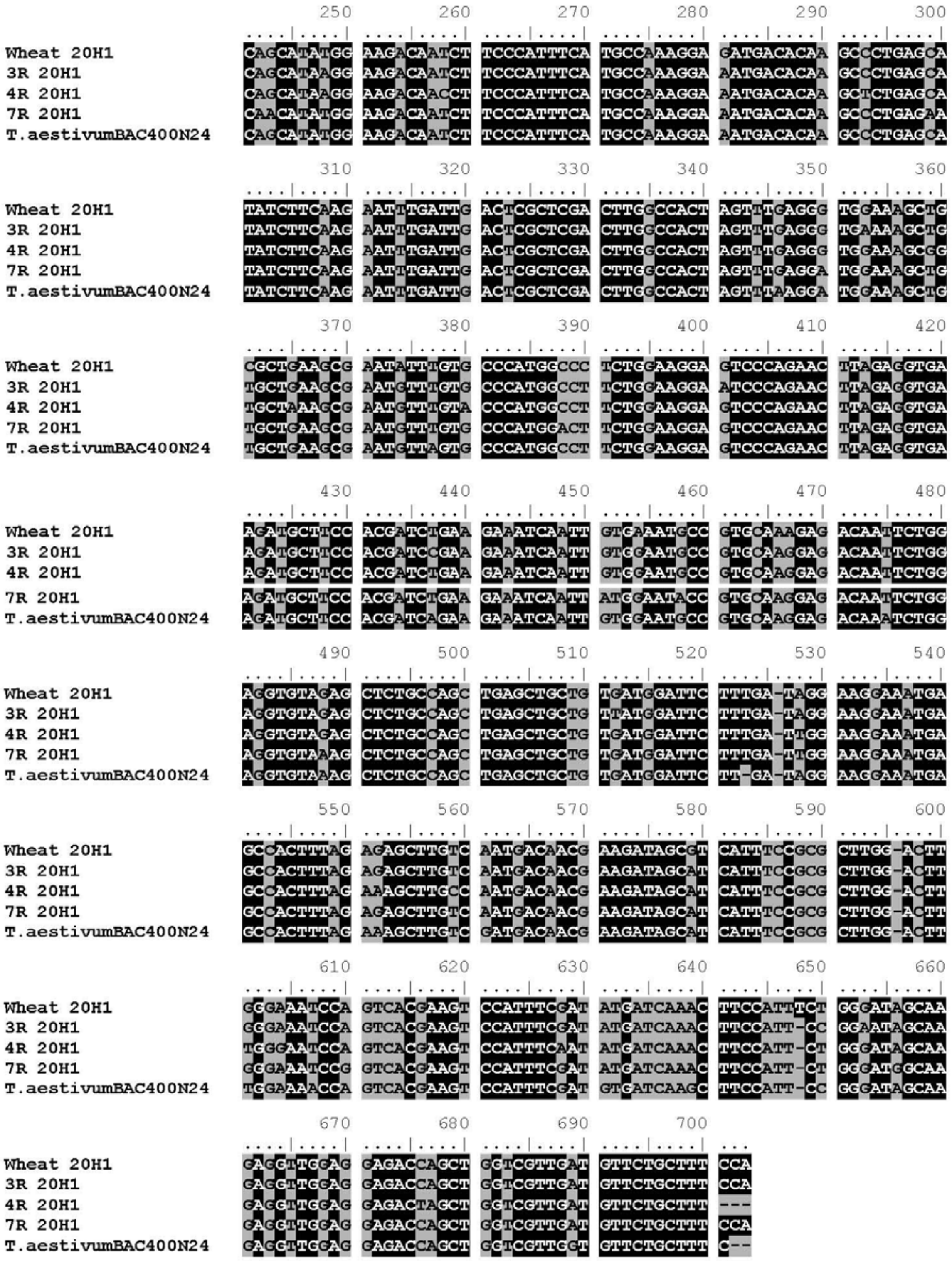

© 2012 by the authors; licensee MDPI, Basel, Switzerland. This article is an open access article distributed under the terms and conditions of the Creative Commons Attribution license (http://creativecommons.org/licenses/by/3.0/).
